# Effect of Cinnamaldehyde on Morphological Alterations of *Aspergillus ochraceus* and Expression of Key Genes Involved in Ochratoxin A Biosynthesis

**DOI:** 10.3390/toxins10090340

**Published:** 2018-08-22

**Authors:** Limin Wang, Jing Jin, Xiao Liu, Yan Wang, Yang Liu, Yueju Zhao, Fuguo Xing

**Affiliations:** Institute of Food Science and Technology, Chinese Academy of Agricultural Sciences/Key Laboratory of Agro-Products Quality and Safety Control in Storage and Transport Process, Ministry of Agriculture, Beijing 100193, China; caassolar@163.com (L.W.); jinjing@caas.cn (J.J.); youcaoxiao@163.com (X.L.); wangyan@caas.cn (Y.W.); zhaoyueju@caas.cn (Y.Z.)

**Keywords:** ochratoxin A, *Aspergillus ochraceus*, cinnamaldehyde, gene expression, real-time PCR

## Abstract

Ochratoxin A (OTA) is a potent nephrotoxic, hepatotoxic, and teratogenic compound which is a significant mycotoxin contaminates cereals during storage. *Aspergillus ochraceus* is the most common producer of OTA in cereals and cereal-derived products. Cinnamaldehyde is a natural substance derived from plant cinnamon playing an important role in the reduction of OTA contamination. In this study, the antifungal and antitoxigenic effect of cinnamaldehyde was investigated with its mechanisms of inhibition of fungal growth at the morphological and ultrastructural levels, and inhibition of OTA biosynthesis at the transcriptional level. Significant *A. ochraceus* growth was inhibited at 0.4–1.6 mmol/L with fumigation. *A. ochraceus* exposed to 0.4 mmol/L of cinnamaldehyde indicated irreversible harmful morphological and ultrastructural modifications such as the folding of the cell, the loss of integrity of the cell wall, the disruption of plasma membrane, the destruction of the mitochondria, and the absence of intracellular organelles. These alterations may be attributed to its inhibition of enzymatic reactions that regulate cell wall synthesis, thus disturbing the morphogenesis and growth of *A. ochraceus*. In the presence of cinnamaldehyde, the tested biosynthetic and regulatory genes like *pks*, *nrps*, *veA*, *laeA* and *velB* were highly downregulated. Moreover, the downregulation effect of cinnamaldehyde increased proportionally with the concentrations. These results suggest that the decrease of OTA production by cinnamaldehyde is attributed to the downregulation of the transcriptional levels of OTA biosynthetic and regulatory genes besides the inhibition of fungal growth. The study reveals the mechanisms of the antifungal and antitoxigenic activities of cinnamaldehyde against *A. ochraceus*, and further emphasizes that cinnamaldehyde could be a safe and effective natural agents against OTA contamination during cereals storage.

## 1. Introduction

Ochratoxin A (OTA) is an important mycotoxin mainly produced by the species of the genera *Aspergillus* and *Penicillium* during pre-harvest period and storage [1]. OTA contaminates a wide range of foods and feeds, including cereals and cereal-derived products, peanuts, oilseeds, coffee beans, grapes, beverages, dried fruits, spices, beer, and wine [2,3,4,5]. OTA is highly nephrotoxic, hepatotoxic, teratogenic, neurotoxic, embryotoxic, genotoxic, and immunosuppressive in nature [5,6,7,8,9], and has been classified as potential carcinogen (group 2B) by the International Agency for Research on Cancer [10]. Therefore, the European Union set the limitation of 5 µg/kg OTA in cereal grains [11], and a similar standard is maintained in China [12]. The main OTA-producing fungi include *Aspergillus ochraceus*, *Aspergillus carbonarius*, *Aspergillus niger*, *Aspergillus westerdijkiae*, *Penicillium nordicum*, and *Penicillium verrucosum* [9,13]. Of them, *A. ochraceus* and *P. verrucosum* are mainly responsible for OTA contamination in wheat, barley, rice, oats, and coffee beans, while other species are mainly responsible for OTA in grapes, raisins, beverages, and wine [9,13,14,15].

To remove OTA in our food chain, numerous approaches have been used to either prevent OTA-producing fungi or block OTA production [9]. Chemical-based fungicides such as low molecular weight organic acids, aromatic hydrocarbons, benzimidazole, and sterol biosynthesis inhibitors are often used to control the post-harvest contamination of mycotoxins in foods [9]. However, many disadvantages are associated with their use, such as the increased risk of toxic residues in foods and fungicide resistance [16,17,18,19]. So, in recent years, the worldwide tendency is to limit chemical fungicide use in grains and foodstuffs [20]. Essential oils extracted from plants have been attractive in both academia and the food industry due to their antimicrobial and antioxidative properties [21]. In our previous study, the inhibitory effect of 10 essential oils on *A. ochraceus* growth and OTA production was investigated using fumigation and contact assays, and cinnamaldehyde proved to be most effective compared with other essential oils, followed by citral and eugenol [9].

To date, the molecular mechanism of action behind cinnamaldehyde inhibits OTA production has not been revealed. Previous studies have showed that several enzymes, such as polyketide synthase (PKS), nonribosomal peptide synthase (NRPS), cytochrome p450 monoxygenase, and halogenase, are involved in the key steps of the OTA biosynthesis [22,23,24]. Based on the results of previous studies and our work, we have determined the steps of OTA biosynthesis and proposed an OTA biosynthetic pathway [25]. In the OTA biosynthetic pathway, *pks* encodes a polyketide synthase which is responsible for the synthesis of 7-methylmellein, the first step of the putative pathway [26], and the *nrps* encodes a nonribosomal peptide which combines OTβ and L-β-phenylalanine to form an amide bond to synthesize OTB [25]. OTA biosynthesis is also associated with genes encoding the velvet regulating proteins (VelB, VeA, and LaeA). *VelB*, *veA*, and *laeA* are transcriptional factors which can coordinate fungal development and secondary metabolism and can activate OTA production [27].

In the present study, to reveal the inhibitory mechanism of *A. ochraceus* growth by cinnamaldehyde, the effect of cinnamaldehyde on *A. ochraceus* hyphae ultrastructure alterations was investigated using scanning electron microscopy (SEM) and transmission electron microscopy (TEM). In order to uncover the molecular mechanism of action by which cinnamaldehyde inhibits OTA biosynthesis, the transcript levels of key OTA biosynthetic and regulatory genes (*pks*, *nrps*, *veA*, *laeA*, and *velB*) were evaluated using real-time PCR.

## 2. Results

### 2.1. Inhibitory Effect of Cinnamaldehyde on the Growth and Ochratoxin A Production by A. ochraceus with Fumigation

**Figure 1:**
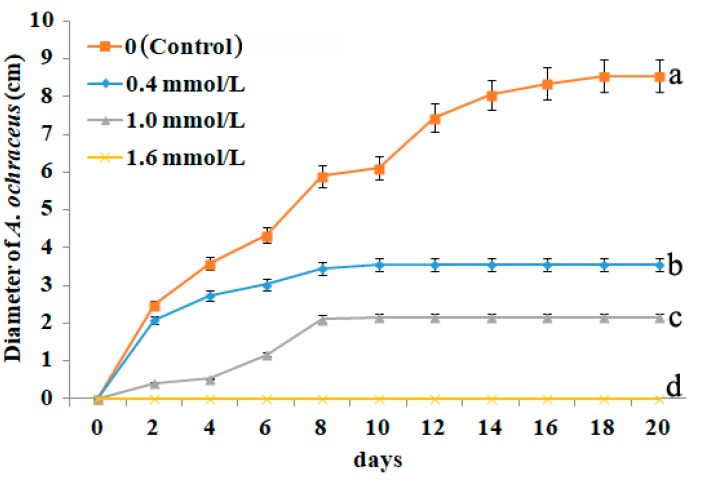
Effect of cinnamaldehyde fumigation on the gowth of *A. ochraceus*. Values represent the means of ten replicates and their standard deviation. Different letters show the significance of difference (*p* < 0.05) between treatments.

The effect of cinnamaldehyde on *A. ochraceus* growth and OTA production were shown in Figure 1 and Table 1, respectively. Cinnamaldehyde could significantly inhibit *A. ochraceus* growth and OTA production at the tested concentrations (0.4–1.6 mmol/L). The inhibitory effect of fungal growth proportionally increased with cinnamaldehyde in concentrations, and also had impact based on the incubation time. The increase in the concentration of cinnamaldehyde (0.4, 1.0, and 1.6 mmol/L) caused a delay in conidia germination and showed higher inhibitory effects. After one day of exposition to 1.6 mmol/L of cinnamaldehyde, the *A. ochraceus* growth was completely inhibited. At the concentration of 0.4 and 1.0 mmol/L, cinnamaldehyde limited the mycelia growth to 3.45 ± 0.10 cm and 2.15 ± 0.05 cm from 8 to 20 days, respectively, and as shown in Table 1, OTA production declined from 1.90 to 0–0.34 ng/mm^2^ colony with the inhibition rate ranging from 82.0% to 100%.

### 2.2. Effect of Cinnamaldehyde on the Morphology of A. ochraceus by SEM

**Figure 2:**
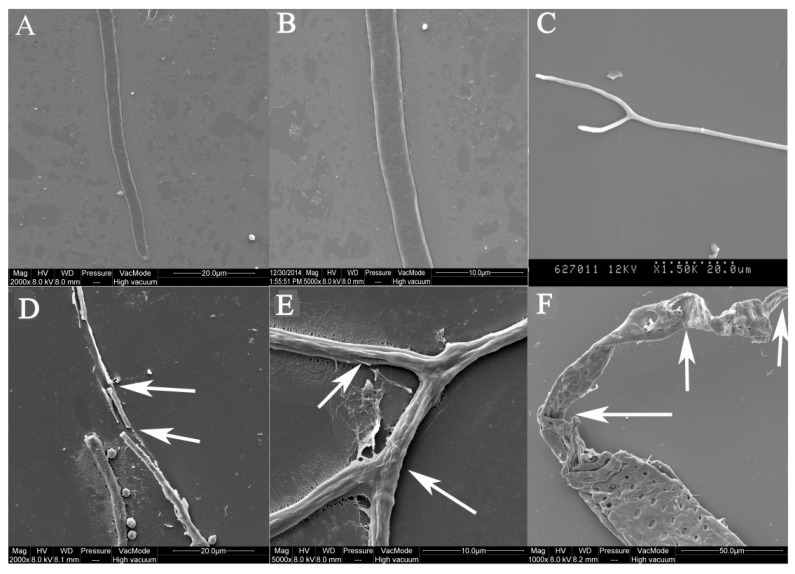
SEM images of the nonfumigated *A. ochraceus* mycelia (**A**–**C**) and fumigated mycelia (**D**–**F**) with 0.4 mmol/L of cinnamaldehyde.

The morphologic alterations of *A. ochraceus* treated with 0.4 mmol/L of cinnamaldehyde are shown in Figure 2. Compared with controls, mycelia exposed to cinnamaldehyde revealed marked alterations in both the whole length of the hyphae and the apical regions. For the *A. ochraceus* that were untreated, the mycelia showed a normal morphology and the hyphae were linear, regular, and homogenous, and their cell walls were smooth (Figure 2A–C). However, this morphology underwent alterations upon exposure to 0.4 mmol/L of cinnamaldehyde. It was noted that the mycelia tips were elongated after cinnamaldehyde treatment and became easy to break (Figure 2D). These hyphae shrank and underwent winding (Figure 2E), then lost their linearity with some depressions on the hyphae surface (Figure 2D,E). The craters were obvious on the cell wall and damages with clear disruption were observed in the cell wall (Figure 2F).

### 2.3. Effect of Cinnamaldehyde on the Ultrastructure of A. ochraceus by TEM

**Figure 3:**
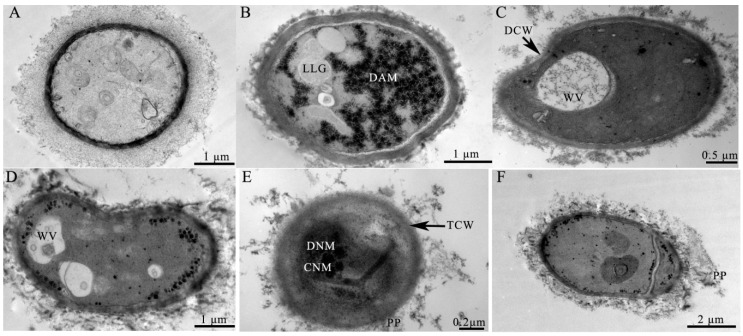
TEM images of *A. ochraceus* cells grown for 24 h in MEA with (**A**) 0 (control), or (**B–F**) 0.4 mmol/L of cinnamaldehyde. DCW, disruption of cell walls; WV, wide vacuoles; DAM, disorganized aggregated mitochondria; LLG, large lipid globules; TCW, thin cell wall; DNM, disintegration of nuclear membrane; CNM, clumping of nuclear material; PP, appearing of the precipitates.

The ultrastructural alterations of *A. ochraceus* treated with 0.4 mmol/L of cinnamaldehyde are shown in Figure 3. The normal ultrastructure of *A. ochraceus* cells showed intact mycelia with sound and homogeneous structure, mitochondria, endoplasmic reticulum, and vacuoles were observed in the cytoplasm obtained by TEM. However, the healthy ultrastructure of *A. ochraceus* cells was disrupted after treated with cinnamaldehyde. The pivotal alterations include leakage of cytoplasmic contents and disruption of cell walls (DCW) (Figure 3B,C). In the cytoplasm appeared wide vacuoles (WV), with disorganized aggregated mitochondria (DAM) and large lipid globules (LLG) (Figure 3B–D). Alterations in the cell wall were also noted including thin cell walls (TCW), disintegration of nuclear membrane (DNM), clumping of nuclear material (CNM) (Figure 3B,E), and even appearing of the precipitates (PP) in the exterior part of the plasmalemma (Figure 3E,F).

### 2.4. Effect of Cinnamaldehyde on Ochratoxin A Biosynthetic and Regulatory Genes Expression

**Figure 4:**
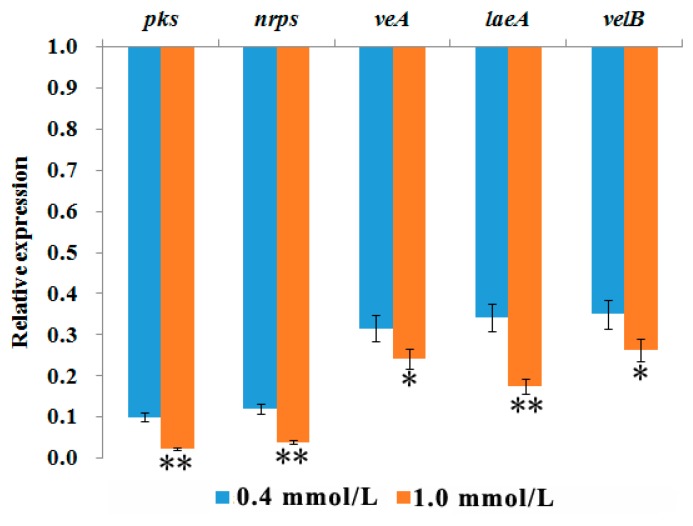
Effect of cinnamaldehyde on the transcript levels of *pks*, *nrps*, *veA*, *laeA*, and *velB* in *A. ochraceus*. Fold change levels of genes in *A. ochraceus* treated with 0.4 mmol/L cinnamaldehyde (blue bars), 1.0 mmol/L cinnamaldehyde (orange bars). Baseline represents the expression levels of genes in untreated fungus (treated as 1.0), * *p* < 0.05, ** *p* < 0.01.

The expression levels of *pks*, *nrps*, *veA*, *laeA*, and *velB* genes in *A. ochraceus* treated with 0.4 and 1.0 mmol/L of cinnamaldehyde were shown in Figure 4. Compared to the control, all the genes were downregulated by cinnamaldehyde. Among the five genes, the transcription level of *pks* gene had the highest downregulation with an average of 98% reduction at 1.0 mmol/L, followed by *nrps*, *laeA*, *veA*, and *velB* with the average of 96%, 84%, 76%, and 74% reduction, respectively. The downregulation effect of cinnamaldehyde proportionally increased with the concentrations. For 0.4 mmol/L of cinnamaldehyde, the downregulation rates of *pks*, *nrps*, *laeA*, *veA*, and *velB* were 90%, 88%, 68%, 66%, and 65%, respectively. These results suggest that cinnamaldehyde causes the down regulation of the tested OTA biosynthetic and regulatory genes, which in turn results in the reduction (82%) of OTA production at 7 days after treatment.

## 3. Discussion

Food and feed contaminated with OTA produced by *A. ochraceus* during storage is a serious global threat to food safety. Consumption of food and products contaminated with OTA causes increased risks of diseases like renal adenomas and carcinomas, hepatocellular carcinomas, Alzheimer’s, and Parkinson’s, which in turn result in severe damage on the health of humans and animals. Multiple approaches have been developed to prevent *A. ochraceus* growth and to control OTA production in food and feed. Antifungal chemicals potentially increase the risk of toxic residues in food and feed and may lead to antifungal-resistant fungus. Therefore, numerous plant essential oils, which are safer, are regarded as alternatives to chemical fungicides and preservatives [28]. The findings of previous studies for natural substance had revealed the inhibitory role of essential oils on different fungal and bacterial species [9,21,28,29,30], and cinnamon oil was highly effective against all the tested organisms and phage [28,29]. In our previous study, cinnamaldehyde, which is the main component of cinnamon oil, was the most effective against *A. ochraceus* growth and OTA production, followed by citral and eugenol [9]. In general, cinnamaldehyde is regarded as safe and widely used as flavor for ingredients in the food industry. Several studies also had investigated the antifungal and antioxidant effects of cinnamaldehyde and reported its activities inhibiting mycelia growth and mortality accompanied by membrane damage [31,32,33]. Earlier studies indicated that cinnamaldehyde had remarkable antimicrobial efficacy on food-borne microorganisms [34,35]. In order to promote the application of cinnamaldehyde in stored foods and feeds, the inhibitory mechanism of *A. ochraceus* growth and OTA production by cinnamaldehyde was investigated in the present study.

With regard to the viability and morphological changes of *A. ochraceus* exposed to cinnamaldehyde, the SEM and TEM images of fungus treated with cinnamaldehyde for 24 h showed marked alterations compared to the controls. The mycelia exhibited obvious alterations in both the length of the hyphae and the apical regions, as well as significant decrease or absence of cytoplasmic contents, a reduction in membrane integrity, the destruction of mitochondria, the disruption of plasma membrane, loss of integrity of the cell wall, and folding of the cell. These results were similar to those reported by Tyagi et al. [36], who found that yeast cells shrank and were obviously absent of cytoplasmic contents after treatment with *Cymbopogon citratus* essential oil. Similarly, previous studies [28,30] found that *Fusarium verticillioides* cells shrank and were obviously absent of cytoplasmic contents after treatment with *Zingiber officinale* essential oil and cinnamaldehyde, respectively.

In *A. ochraceus* treated with cinnamaldehyde, the loss of cytoplasm, the destruction of mitochondria, the disruption of plasma membrane, the loss of integrity of the cell wall, and the folding of the cell were observed. Similarly, the decreased diameter and the thinning of the hyphae wall were observed in *A. niger* treated with *Cymbopogon nardus* (L) essential oils [37]. After treatment with *Thymus eriocalyx* and *T. x-porlock* essential oils, the reduced diameter and thickness of the hyphal wall were also observed in *A. niger* [21]. Furthermore, ultrastructural analysis highlighted that the multiple effects of essential oils on *Aspergillus fumigatus*, *Trichophyton rubrum*, and *F. verticillioides* cells, were damage to the cell walls, membranes, cytoplasmic contents, and decreasing in elastase and keratinase activities [28,38]. Cinnamaldehyde has also been reported to inhibit the activities of chitin synthase 1 and β-(1, 3)-glucan synthase, which are key cell wall synthesizing enzymes in fungi [39]. In human promyelocytic leukemia HL-60 cells, cinnamaldehyde also affected cell wall-synthesizing enzymes or mitochondria [40]. These modifications in the cell wall may be due to the lipophilic properties of essential oils, which making them pervious to the cytoplasmic membrane and cell wall, thus increasing the possibility of their interaction with the cytoplasmic membrane and cell wall, inhibiting cell wall-synthesizing enzymatic reactions, and disrupting cell integrity [21,33]. This mechanism of action disrupts membrane integrity and fluidity, alters the structure of several layers of phospholipids, polysaccharides, proteins, and fatty acids, and in turn results in the leakage of cytoplasmic contents [28].

Complete inhibition of *A. ochraceus* growth and OTA production were observed when 1.6 mmol/L of cinnamaldehyde was applied. However, a significant reduction (82%) in the OTA production with less inhibition in *A. ochraceus* growth (41%) was observed at cinnamaldehyde concentration of 0.4 mmol/L. This result suggests that OTA reduction may be due to the downregulation of OTA biosynthetic and regulatory genes. Several natural compounds have been proved to inhibit mycotoxins production by down regulating the transcript levels of biosynthetic genes [9]. For example, curcumin inhibited AFB_1_ production in *A. parasiticus* by reducing the transcript levels of aflatoxin biosynthetic genes like *pksA*, *ver-1*, *nor-1*, *omtA*, and *aflR* [41,42]; cinnamaldehyde, eugenol, and citral inhibited AFB_1_ production in *A. flavus* by reducing the transcript levels of *ver-1*, *nor-1*, *omtA*, *aflR*, and *aflT* [42]; 2-phenylethanol reduced the expression of the structural genes (*aflC*, *aflD*, *aflO*, and *aflM*) in aflatoxin pathway [43]; *Zataria multiflora* Boiss essential oil reduced the transcript levels of *aflD*, *aflM*, and *aflP* in *A. paraciticus* [44]; piperine inhibited AFB_1_ production in *A. flavus* by downregulating the expression of almost all genes participating in aflatoxin biosynthetic pathway [45]; and eugenol inhibited AFB_1_ production in *A. flavus* by reducing expression of 20 of 29 genes in aflatoxin biosynthetic pathway using RNA-seq [46]. In this study, *pks*, *nrps*, *veA*, *laeA*, and *velB* genes, which are involved in OTA biosynthesis, were highly downregulated by cinnmaldehyde at 0.4 and 1.0 mmol/L. A similar result was observed previously [41,42,43,44,45,46] in *A. parasiticus* and *A. flavus*. The *pks* gene had the highest downregulation, followed by *nrps*, *laeA*, *veA*, and *velB*. The *pks* and *nrps* genes are included in the OTA-putative gene cluster [25,47,48], while, *velA*, *laeA*, and *velB* are known as transcription factors regulating the secondary metabolism [27,49]. The downregulation of *pks* and *nrps* was significant higher than other three genes. These results might be explained based on the role described for VeA and LaeA in other fungi. Previous studies indicated that the complex formed by VelB, VeA, and LaeA proteins connects light-response with fungal development and secondary metabolism, including the positive regulation of the OTA biosynthetic genes’ expression [26,27]. These results suggest that cinnamaldehyde can suppress the transcription of genes such as *veA*, *laeA*, *velB*, *pks*, and *nrps*, and in turn down regulates both fungal development and OTA biosynthesis.

The results revealed the inhibitory mechanism of *A. ochraceus* growth and OTA production by cinnamaldehyde. Cinnamaldehyde causes irreversible harmful morphological and ultrastructural changes, and the downregulation of OTA biosynthetic and regulatory genes, which in turn results in the inhibition of fungal growth and OTA production. These findings further emphasize the toxicity of cinnamaldehyde on fungi and mean that it is a good alternative to chemical fungicides and preservatives during food and feed storage.

## 4. Materials and Methods

### 4.1. Cinnamaldehyde and Ochratoxin A Standards

The cinnamaldehyde (96%) used in the experiments was purchased from Jiangxi Xuesong (Jiangxi Xue Song Natural Medicinal Oil Co., Ltd., Ji’an, Jiangxi, China) and its concentration was confirmed in the lab by Gas Chromatography-Mass Spectrometer (GC-MS, Thermo Fisher Scientific, Waltham, MA, USA). 100 μg/mL of OTA (Sigma Chemical, St. Louis, MO, USA) was prepared in acetonitrile:water (50:50, *v*/*v*) and stored in amber vials at −18 °C.

### 4.2. Fungal Strain and Culture Conditions

*A. ochraceus* fc-1 is a high OTA-producing strain which is maintained in our lab. The strain was grown on potato dextrose agar (PDA) and stored at 4 °C. Conidia suspensions were harvested by surface washing of 7-day-old *A. ochraceus* PDA cultures with 0.1% tween-80 in sterile deionized water. Then the conidia were counted using a hemocytometer and adjusted to 1 × 10^6^ conidia/mL with 0.1% of Tween-80 solution.

### 4.3. Effect of Cinnamaldehyde on Fungal Growth and Ochratoxin A Production

To evaluate the inhibitory effect of cinnamaldehyde on fungal growth and OTA production, the treatment of *A. ochrceus* with cinnamaldehyde by fumigation was performed according to the method described by [28,50] with minor modifications. Briefly, 10 µL of 10^6^ conidia/mL were spotted in the center of Petri dishes containing 20 mL of MEA. Each liter of MEA medium contained 30.0 g malt extract, 3.0 g soy peptone, and 15.0 g agar. The pH of the medium was adjusted to 5.6 ± 0.2. Subsequently, 0, 1, 2.5, and 4 µL of cinnamaldehyde (96%) were added to a 5-mm diameter sterile filter paper disk which was placed on the medium-free cover of the dish for each Petri dish, to the final concentrations of 0, 0.4, 1.0, and 1.6 mmol/L, respectively. According to our measurement, the empty space of each dish with MEA medium is 20 mL; the above concentrations were obtained after full fumigation of cinnamaldehyde in the sealed dish. The sealed dishes were incubated at 28 ± 1 °C for 20 days. All treatments were repeated 5 times and the experiment was conducted twice. The average diameters were obtained by measuring fungal colony in two directions at right angles to each other every 2 days [28].

### 4.4. The Extraction and Determination of Ochratoxin A

OTA production on MEA was assessed according to a method described previously [51,52] with the following modifications. After incubation, three agar plugs (6 mm diameter) were removed starting from the center to the edge of the colony each (4.5 cm), placed in a 1.5 mL glass vial, and then stored at −20 °C until extraction. The three plugs were mixed with 1 mL methanol, vortexed and kept at room temperature (about 20 °C) for 1 h, mixed again, and filtered using 0.22 µm disk filters for high-performance liquid chromatography (HPLC) analysis of OTA.

OTA in culture extracts was detected and quantified using a HPLC unit according to the methodology proposed by [9,53] with minor modifications. Waters 2695 HPLC (Waters Corporation, Milford, MA, USA) with a 2475 fluorescence detector (λ_exc_ 330 nm; λ_em_ 460 nm) and a Agilent TC-C18 column (250 × 4.6 mm, 5 µm) was applied. At room temperature, samples (50 µL) were injected, then the mobile phase (acetonitrile/water/acetic acid, 99:99:2, *v/v/v*) [5] was pumped at a flow rate 1.0 mL/min. The retention time was around 6 min. The calibration curve was obtained using 5, 25, 50, 75, and 100 ng/g OTA standards. The OTA concentration was determined by comparing peak areas of sample extracts with the calibration curve. Mean recovery was measured by spiking the MEA medium with OTA standards at 5, 25, 50, 75, and 100 ng/g and was estimated at 89.2 ± 9.7%. The limit of detection was 1 ng/g.

### 4.5. Scanning Electron Microscopy (SEM) and Transmission Electron Microscopy (TEM)

SEM was operated using a method proposed previously [28] with minor modifications. The mycelia of *A. ochraceus* untreated and treated with 0.4 mmol/L cinnamaldehyde were collected from MEA cultures and mixed with formaldehyde, subsequently washed and dehydrated using PBS buffer and gradient ethanol solutions, respectively. Then, the samples were suffered from critical-point drying in CO_2_ with a method described previously [28,54] and sputter-coated with gold. For the SEM analysis, the mycelia were fixed onto stubs, then placed on the gold coater holder and coated with a 1.40 nm layer of gold [28]. TEM was carried out using a method reported previously [28]. The *A. ochraceus* mycelia, both untreated and treated with cinnamaldehyde, were observed under a Hitachi H-7500 TEM.

### 4.6. Real-Time PCR Analysis of OTA Biosynthetic and Regulatory genes

To evaluate the effect of cinnamaldehyde (0.4 and 1.0 mmol/L) on the transcript levels of OTA biosynthetic and regulatory genes, a 10 µL conidia suspension (10^6^ conidia/mL) was spotted in the center of MEA medium covered with sterile cellophane layers. After incubations for 7 days, *A. ochraceus* mycelia were collected from cellophane layers and flash-frozen using liquid nitrogen and subsequently ground to fine powder for RNA isolation. Total RNA was extracted from 100 mg of mycelia using the Fungal RNA Kit (Omega Bio-Tek, Norcross, GA, USA) according to the manufacturer’s instructions. RNA samples were treated with RNase-Free DNase (QIAGEN GmbH, Hilden, Germany) to digest the residual DNA. The purity and concentrations of RNA were detected by measuring the absorbance of RNA samples at 260 and 280 nm using a NanoDrop 2000 (Thermo Fisher Scientific, Wattham, MA, USA). The cDNA was synthesized by reverse transcription from 5 μg of total RNA using a Takara RNA PCR Kit (AMV) ver. 3.0 (Takara Bio Inc, Beijing, China). All primers were designed using Primer Premier 6.0 software (Premier Biosoft International, Palo Alto, CA, USA, 2010) and their specificities were validated using the Primer-BLAST software [55]. All primers were synthesized by Sangon Biotech (Beijing, China) and their sequences are listed in Table 2.

Experiments were carried out using an ABI Prism 7500 system (Applied Biosystems, Foster City, CA, USA). The amplification was performed using the SYBR Green PCR Master Mix (Applied Biosystems, Foster City, CA, USA) and each reaction well contained a final volume of 20 μL mix: 10 μL of SYBR Green PCR Master Mix, 1 μL of cDNA material (100 ng), 0.5 μL of each primer (10 mmol/L), and Nuclease-Free water 8 μL. The thermal protocol was carried out as described previously [56]. The *GADPH* was used as reference gene [25,57]. The results were analyzed using the sequence detection system software 1.9.1 (Applied Biosystems, Foster City, CA, USA) and the relative expression of targeted gene was calculated using the 2^−ΔΔct^ method [58]. Three distinct experiments were conducted, each including at least three biological replicates of each condition.

### 4.7. Statistical Analysis

All the statistical analyses were conducted using SAS v. 9.2 (SAS Institute Inc., Cary, NC, USA, 2007) and Microsoft Excel 2013 (15.0.5059.1000, Microsoft Corporation, Redmond, WA, USA, 2013). The gene expression analyses were evaluated by one-way analysis of variance (ANOVA, SAS v. 9.2, SAS Institute Inc., Cary, NC, USA, 2013). Mean comparisons were analyzed by Turkey’s multiple range tests. Differences were considered to be significant at *p* < 0.05.

## Figures and Tables

**Table 1:** Inhibitory effect of cinnamaldehyde on *Ochratoxin A* production.

Concentration of Cinnamaldehyde (mmol/L)	OTA Production (ng/mm^2^ colony)	Inhibition Ratio (%)
0	1.90 ± 0.45 a	-
0.4	0.34 ± 0.14 b	82.0
1.0	0.03 ± 0.03 c	98.7
1.6	0.00 ± 0.00 d	100.0

Values are mean (*n* = 10) ± RSD (relative standard deviation). Different letters show the significance of differences (*p* < 0.05) between treatments.

**Table 2:** Primers sequences and their PCR products.

Gene	Primer Name	Primers (5′ to 3′)	Product Length (bp)
*GADPH*	G-F G-R	CGCTCAGAACATCATCCCCA ATGTCCTCGTAGGTGACGGA	142
*pks*	P-F P-R	CGCCTCATCATCAATCCTT CAACTCGGTCAAGCAGAT	144
*nrps*	N-F N-R	TGTGGACATCTGGAAGCA GTGAACGAGGTGAATTGGA	136
*veA*	VA-F VA-R	ACCAACATCAGCCGTGTCAT GTACGAGTCAGGCGTGGAAA	159
*laeA*	L-F L-R	GCCCAATAGCCCACAACTCT TGTACCACCGAGCAACCTTC	141
*velB*	VB-F VB-R	TACTATTCGGGAGGCGGTCA TTGTTGTCGGGATCGGTCAG	143

## References

[1] Valero A., Farré J.R., Sanchis V., Ramos A.J., Marín S. (2006). Effects of fungal interaction on ochratoxin A production by *A. carbonarius* at different temperatures and *a*_w_. Int. J. Food Microbiol..

[2] Jørgensen K. (1998). Survey of pork, poultry, coffee, beer and pulses for ochratoxin A. Food Addit. Contam..

[3] Magnoli C., Astoreca A., Ponsone L., Combina M., Palacio G., Rosa C.A.R., Dalcero A.M. (2004). Survey of mycoflora and ochratoxin A in dried vine fruits from Argentina markets. Lett. Appl. Microbiol..

[4] Magnoli C., Hallak C., Astoreca A., Ponsone L., Chiacchiera S.M., Palacio G., Dalcero A. (2005). Surveillance of toxigenic fungi and ochratoxin A in feedstuffs from Córdoba Province, Argentina. Vet. Res. Commun..

[5] Magnoli C., Astoreca A., Ponsone M.L., Fernández-Juri M.G., Barberis C., Dalcero A.M. (2007). Ochratoxin A and *Aspergillus* section *Nigri* in peanut seeds at different months of storage in Córdoba, Argentina. Int. J. Food Microbiol..

[6] Denli M., Perez J.F. (2010). Ochratoxins in feed, a risk for animal and human health: Control strategies. Toxins.

[7] Pfohlleszkowicz A. (2009). Ochratoxin A and aristolochic acid involvement in nephropathies and associated urothelial tract tumours. Arch. Ind. Hyg. Toxicol..

[8] Ringot D., Chango A., Schneider Y.J., Larondelle Y. (2006). Toxicokinetics and toxicodynamics of ochratoxin A, an update. Chem.-Biol. Interact..

[9] Hua H., Xing F., Selvaraj J.N., Wang Y., Zhao Y., Zhou L. (2014). Inhibitory effect of essential oils on *Aspergillus ochraceus* growth and ochratoxin A production. PLoS ONE.

[10] Harnden D.G. (1977). Iarc monographs on the evaluation of carcinogenic risk of chemicals to man. Vol. 10: Some naturally occurring substances. IARC Monogr. Eval. Carcinog. Risks Chem. Hum..

[11] Commission of the European Communities (2010). Commission regulation (EU) No 165/2010 of 26 February 2010 amending, Regulation (EC) No 1881/2006 setting maximum levels for certain contaminants in foodstuffs as regards aflatoxins. Off. J. Eur. Union L50.

[12] (2017). China National Food Safety Standard (GB 2761-2017), Maximum Limit of Mycotoxins in Food. http://www.nhfpc.gov.cn/sps/s7891/201704/b83ad058ff544ee39dea811264878981.shtml.

[13] Sokolić-Mihalak D., Frece J., Slavica A., Delaš F., Pavlović H., Markov K. (2012). The effect of wild thyme (*Thymus Serpyllum* L.) essential oil components against ochratoxin-producing *Aspergilli*. Arch. Ind. Hyg. Toxicol..

[14] Čvek D., Markov K., Frece J., Dragičević T., Majica M., Delaš F. (2010). Growth inhibition of *Aspergillus ochraceus* ZMPBF 318 and *Penicillium expansum* ZMPBF 565 by four essential oils. Arch. Ind. Hyg. Toxicol..

[15] Reddy K.R.N., Salleh B., Saad B., Abbas H.K., Abel C.A., Shier W.T. (2010). An overview of mycotoxin contamination in foods and its implications for human health. Toxin Rev..

[16] Al-Omair A., Helaleh M.I.H. (2004). Selected-Ion storage GC–MS analysis of polycyclic aromatic hydrocarbons in palm dates and tuna fish. Chromatographia.

[17] Chen P.J., Moore T., Nesnow S. (2008). Cytotoxic effects of propiconazole and its metabolites in mouse and human hepatoma cells and primary mouse hepatocytes. Toxicol. In Vitro.

[18] Chilvers M.I., Hay F.S., Hills J., Dennis J.J.C., Wilson C.R. (2006). Influence of benzimidazole fungicides on incidence of *Botrytis allii* infection of onion leaves and subsequent incidence of onion neck rot in storage in Tasmania, Australia. Aust. J. Exp. Agric..

[19] Isaac S. (1999). What is the mode of action of fungicide and how do fungi develop resistance?. Mycologist.

[20] López A.G., Theumer M.G., Zygadlo J.A., Rubinstein H.R. (2004). Aromatic plants essential oils activity on *Fusarium verticillioides* Fumonisin B_1_ production in corn grain. Mycopathologia.

[21] Rasooli I., Rezaei M.B., Allameh A. (2006). Growth inhibition and morphological alterations of *Aspergillus niger* by essential oils from *Thymus eriocalyx* and *Thymus x-porlock*. Food Control.

[22] Gallo A., Bruno K.S., Bruno K.S., Solfrizzo M., Perrone G., Mulè G. (2012). New insight into the ochratoxin A biosynthetic pathway through deletion of a nonribosomal peptide synthetase gene in *Aspergillus carbonarius*. Appl. Environ. Microbiol..

[23] Harris J.P., Mantle P.G. (2001). Biosynthesis of ochratoxins by *Aspergillus ochraceus*. Phytochemistry.

[24] Huff W., Hamilton P. (1979). Mycotoxins-their biosynthesis in fungi: Ochratoxins-metabolites of combined pathways. J. Food Prot..

[25] Wang Y., Wang L., Wu F., Liu F., Wang Q., Zhang X., Selvaraj J.N., Zhao Y., Xing F., Yin W.-B. (2018). A consensus ochratoxin A biosynthetic pathway: Insights from the genome sequence of *Aspergillus ochraceus* and a comparative genomic analysis. Appl. Environ. Microbiol..

[26] Wang L., Wang Y., Wang Q., Liu F., Selvaraj J.N., Liu L. (2015). Functional characterization of new polyketide synthase genes involved in ochratoxin A biosynthesis in *Aspergillus ochraceus* fc-1. Toxins.

[27] Crespo-Sempere A., Marín S., Sanchis V., Ramos A.J. (2013). *VeA* and *LaeA* transcriptional factors regulate ochratoxin A biosynthesis in *Aspergillus carbonarius*. Int. J. Food Microbiol..

[28] Xing F., Hua H., Selvaraj J.N., Zhao Y., Zhou L., Liu X., Liu Y. (2014). Growth inhibition and morphological alterations of *Fusarium verticillioides* by cinnamon oil and cinnamaldehyde. Food Control.

[29] Chao S.C., Young D.G., Oberg C.J. (2000). Screening for inhibitory activity of essential oils on selected bacteria, fungi and viruses. J. Essent. Oil Res..

[30] Yamamoto-Ribeiro M.M.G., Crespan R., Kohiyama C.Y., Ferreira F.D., Mossini S.A.G., Silva E.L. (2013). Effect of *Zingiber officinale* essential oil on *Fusarium verticillioides* and fumonisin production. Food Chem..

[31] Molania T., Moghadamnia A.A., Pouramir M., Aghel S., Moslemi D., Ghassemi L., Motallebnejad M. (2012). The effect of cinnamaldehyde on mucositis and salivary antioxidant capacity in gamma-irradiated rats (a preliminary study). DARU J. Pharm. Sci..

[32] Taquchi Y., Hayama K., Okada M., Sagawa T., Arai R., Abe S. (2011). Therapeutic effects of cinnamaldehyde and potentiation of its efficacy in combination with methylcellulose on murine oral candidiasis. Med. Mycol. J..

[33] Taguchi Y., Hasumi Y., Abe S., Nishhiyama Y. (2013). The effect of cinnamaldehyde on the growth and the morphology of *Candida albicans*. Med. Mol. Morphol..

[34] Hong D., Han M. (2013). Crystal structure of catena-bis (µ3-5-methoxyisophthalato)-bis(µ2-1,6-bis (imidazol-1-yl)-hexane) nickel (II), [Ni-2(CH_3_OC_8_H_3_O_4_)2(C_12_H_18_N_4_)_2_], C_42_H_48_N_8_Ni_2_O_10_. Z. Krist-New Cryst. St..

[35] Visvalingam J., Holley R.A. (2012). Temperature-dependent effect of sublethal levels of cinnamaldehyde on viability and morphology of *Escherichia coli*. J. Appl. Microbiol..

[36] Tyagi A.K., Malik A. (2009). Liquid and vapour-phase antifungal activities of selected essential oils against *Candida albicans*: Microscopic observations and chemical characterization of *Cymbopogon citratus*. BMC Complement. Altern. Med..

[37] De Billerbeck V.G., Roques C.G., Bessiére J.M., Fonvieille J.L., Dargent R. (2001). Effects of *Cymbopogon nardus* (L.) W. Watson essential oil on the growth and morphogenesis of *Aspergillus niger*. Can. J. Micro Biol..

[38] Khan M.S.A., Ahmad I. (2011). In vitro antifungal, anti-elastase and anti-keratinase activity of essential oils of Cinnamomum-, Syzygium- and Cymbopogon-species against *Aspergillus fumigatus* and *Trichophyton rubrum*. Phytomedicine.

[39] Bang K.H., Lee D.W., Park H.M. (2000). Inhibition of fungal cell wall synthesizing enzymes by trans-cinnamaldehyde. Biosci. Biotech. Biochem..

[40] Ka H., Park H.J., Jung H.J., Choi J.W., Cho K.S., Ha J. (2003). Cinnamaldehyde induces apoptosis by ROS-mediated mitochondrial permeability transition in human promyelocytic leukemia HL-60 cells. Cancer Lett..

[41] Jahanshiri Z., Shams-Ghahfarokhi M., Allameh A., Razzaghi-Abyaneh M. (2012). Effect of curcumin on *Aspergillus parasiticus* growth and expression of major genes involved in the early and late stages of aflatoxin biosynthesis. Iran. J. Public Health.

[42] Liang D., Xing F., Selvaraj J.N., Liu X., Wang L., Hua H., Zhou L., Zhao Y., Wang Y., Liu Y. (2015). Inhibitory effect of cinnamaldehyde, citral and eugenol on aflatoxin biosynthetic gene expression and aflatoxin B_1_ biosynthesis in *Aspergillus flavus*. J. Food Sci..

[43] Hua S.S.T., Beck J.J., Sarreal S.B.L., Gee W. (2014). The major volatile compound 2-phenylethanol from the biocontrol yeast, *Pichia anomala*, inhibits growth and expression of aflatoxin biosynthetic genes of *Aspergillus flavus*. Mycotoxin Res..

[44] Yahyaraeyat R., Khosravi A.R., Shahbazzadeh D., Khalaj V. (2013). The potential effects of *Zataria multiflora* Boiss essential oil on growth, aflatoxin production and transcription of aflatoxin biosynthesis pathway genes of toxigenic *Aspergillus parasiticus*. Braz. J. Microbiol..

[45] Caceres I., Khoury R.E., Bailly S., Oswald I.P., Puel O., Bailly J.-D. (2017). Piperine inhibits aflatoxin B_1_ production in *Aspergillus flavus* by modulating fungal oxidative stress response. Fungal Genet. Biol..

[46] Lv C., Wang P., Ma L., Zheng M., Liu Y., Xing F. (2018). Large-scale comparative analysis of eugenol-induced/repressed genes expression in *Aspergillus flavus* using RNA-seq. Front. Microbiol..

[47] Gerin D., De Miccolis Angelini R.M., Pollastro S., Faretra F. (2016). RNA-Seq reveals OTA-related gene transcriptional changes in *Aspergillus carbonarius*. PLoS ONE.

[48] Gil-Serna J., García-Díaz M., González-Jaén M.T., Vázquez C., Patiño B. (2018). Description of an orthologous cluster of ochratoxin A biosynthetic genes in *Aspergillus* and *Penicillium* species. A comparative analysis. Int. J. Food Microbiol..

[49] Park H.S., Ni M., Jeong K.C., Kim Y.H., Yu J.H. (2012). The role, interaction and regulation of the velvet regulator *VelB* in *Aspergillus nidulans*. PLoS ONE.

[50] Soliman K.M., Badeaa R.I. (2002). Effect of oil extracted from some medicinal plants on different mycotoxigenic fungi. Food Chem. Toxicol..

[51] Leong S.L.L., Hocking A.D., Scott E.S. (2006). Effect of temperature and water activity on growth and ochratoxin A production by Australian *Aspergillus carbonarius* and *A. niger* isolates on a simulated grape juice medium. Int. J. Food Microbiol..

[52] Copetti M.V., Iamanaka B.T., Mororó R.C., Pereira J.L., Frisvad J.C., Taniwaki M.H. (2012). The effect of cocoa fermentation and weak organic acids on growth and ochratoxin A production by *Aspergillus* species. Int. J. Food Microbiol..

[53] Scudamore K.A., Macdonald S.J. (1998). A collaborative study of an HPLC method for determination of ochratoxin A in wheat using immunoaflinity column clean-up. Food Addit. Contam..

[54] Bray D., Williams J.R., Clifford A.A. (2000). Critical point drying of biological specimens for scanning electron microscopy. Supercritical Fluid Methods and Protocols.

[55] Ye J., Coulouris G., Zaretskaya I., Cutcutache I., Rozen S., Madden T.L. (2012). Primer-BLAST: A tool to design target-specific primers for polymerase chain reaction. BMC Bioinf..

[56] Xing F., Wang L., Liu X., Selvaraj J.N., Wang Y., Zhao Y., Liu Y. (2017). Aflatoxin B_1_ inhibition in *Aspergillus flavus* by *Aspergillus niger* through down-regulating expression of major biosynthetic genes and AFB_1_ degradation by atoxigenic *A. flavus*. Int. J. Food Microbiol..

[57] Wang Y., Liu F., Wang L., Wang Q., Selvaraj J.N., Zhao Y., Wang Y., Xing F., Liu Y. (2018). pH-signaling transcription factor *AopacC* regulators ochratoxin A biosynthesis in *Aspergillus ochraceus*. J. Agric. Food Chem..

[58] Livak K.J., Schmittgen T.D. (2001). Analysis of relative gene expression data using real-time quantitative PCR and the 2 ^−ΔΔCT^ method. Methods.

